# In Vivo Antifungal Activity of South African Medicinal Plant Extracts against *Fusarium* Pathogens and Their Phytotoxicity Evaluation

**DOI:** 10.3390/plants9121668

**Published:** 2020-11-27

**Authors:** Hlabana A. Seepe, Kafua E. Lodama, René Sutherland, Winston Nxumalo, Stephen O. Amoo

**Affiliations:** 1 Agricultural Research Council—Vegetables, Industrial and Medicinal Plants Research, Roodeplaat, Private Bag X293, Pretoria 0001, South Africa; LodamaKM@arc.agric.za (K.E.L.); SutherlandR@arc.agric.za (R.S.); 2 Department of Chemistry, University of Limpopo, Private Bag X1106, Sovenga, Polokwane 0727, South Africa; winston.nxumalo@ul.ac.za; 3 Indigenous Knowledge Systems Centre, Faculty of Natural and Agricultural Sciences, North-West University, Private Bag X2046, Mmabatho 2735, South Africa; 4 Department of Botany and Plant Biotechnology, Faculty of Science, University of Johannesburg, P.O. Box 524, Auckland Park 2006, South Africa

**Keywords:** *Fusarium* species, maize seeds, medicinal plant extracts, seed germination, smallholder farmers

## Abstract

Smallholder farmers play a major role in crop production towards household food security, particularly in resource-poor communities. Maize is a common crop produced in smallholder farming and it is cultivated from seeds that has been stored and re-used for years. Spoilage of stored grains is a major challenge, which leads to yield loss and poor seed quality. The objectives of this study were to evaluate in vivo antifungal activity of selected plant extracts against *Fusarium* pathogens on maize seeds, and to evaluate their phytotoxicity on seed germination and seedling growth. Fresh leaves collected from eight medicinal plants were dried and selectively extracted with water, ethyl acetate or acetone. The dried extracts were evaluated for antifungal activity against *Fusarium* pathogens (*F. proliferatum*, *F. oxysporum*, *F. subglutinans*, *F. verticilloides*, *F. semitectum*, *F. chlamydosporum*, *F. solani*, *F. equisite* and *F. graminearum*) inoculated on maize seeds. *Melia azedarach* acetone extract showed strong antifungal activity (97% inhibition) against *F. proliferatum* while combined acetone extracts from *Combretum erythrophyllum* and *Quercus acutissima* exhibited 96%, 67% and 56% inhibition against *F. verticilloides*, *F. proliferatum* and *F. solani*, respectively. With the exception of *Quercus acutissima* ethyl acetate, none of the extracts significantly inhibited seed germination when compared to untreated seeds. This study showed that plant extracts could control *Fusarium* diseases without any adverse effects on maize seed germination or plant growth.

## 1. Introduction

Maize (*Zea mays* L.) is the most important grain crop and dietary staple food in the world [1,2]. It has the potential to alleviate poverty and plays a key role in food security and economic wellbeing particularly in sub-Saharan Africa. In most developing countries, maize is consumed primarily as a porridge and sometimes as freshly boiled or roasted grains. It is also consumed in processed form, such as snacks and cereals. Naturally, it is a source of carbohydrate, fat, fiber, vitamins, macro- and micronutrient elements [3]. In addition to its utilization as feed for livestock and poultry, maize is an excellent source of raw material for many industrial products, such as starch, oil, organic liquids and alcoholic beverages [4,5]. Maize is cultivated in over 48 African countries, with more than 208 million people in sub-Saharan Africa depending on it as a source of food and income [6]. In South Africa, both commercial and smallholder-farming systems are responsible for the production of maize, although this production is dominated by commercial farming. In addition to the availability of cultivated land, commercial farmers rely on irrigation systems, fertilization, pesticides, improved maize cultivars or hybrids and modern machineries to optimize production. In contrast, maize production in smallholder farming is labour-intensive and almost fully rain-fed. Smallholder farmers depend on indigenous knowledge and traditional rudimentary methods to control crop diseases both in the field and during storage.

The smallholder farming system is faced with challenges, including climate change, crop diseases, spoilage of grains during storage and poor seed germination [7]. These challenges, among others, may result in drastic yield loss and an increase in food insecurity and food prices. Nevertheless, smallholder farming remains an important source of food and income generation, particularly in poor rural communities [8,9,10,11]. Successful maize production in smallholder farming is dependent on locally sourced seeds. After harvest, the seeds are stored, exchanged or sold to other community members for consumption or for cultivation during the next planting season. This household-based seed system approach is self-sustainable [12,13]. However, it is threatened by microbial infections that often occur during cultivation, transportation and post-harvest storage [14]. Microbial infections can reduce yield, nutritional value of the seeds and negatively affect seed germination [15].

*Fusarium* species including *F. verticillioides*, *F. subglutinans* and *F. proliferatum* are among spoilage pathogens associated with yield loss in maize production [16,17]. Another major concern is health complications associated with consumption of grains contaminated with mycotoxins produced by these species [18,19,20]. Mycotoxins may cause fungal keratitis, kidney disorders, oesophageal and liver cancer [20,21,22,23,24]. The use of conventional synthetic fungicides as a strategy to control fungi during storage may not be ideal in smallholder farming. These chemicals are largely inaccessible and unaffordable to smallholder farmers. Smallholder farming is practiced in resource-poor communities, where stored grains may be consumed during the storage period. Therefore, seeds or grains treated with synthetic fungicides may cause complications or food poisoning. There is a need to develop relatively cheap and sustainable strategies that can be used to control or reduce grain spoilage, particularly during storage in poor rural communities.

The use of botanicals as an alternative source of bio-pesticides in crop protection has gained momentum because plants are biodegradable and readily available [25,26,27]. Medicinal plant species synthesize different secondary metabolites that perform important biological functions and defend plants against microbes and insects [28,29,30]. Plants have been traditionally used for many years to treat different ailments in both human and domesticated animals, and they are considered to be relatively safe and environmental-friendly [27,31,32]. Despite the importance of maize in smallholder farming systems and the need for alternative forms of crop protection to traditional pesticides, little research has been carried out on bio-pesticides. Therefore, the aim of this study was to evaluate in vivo antifungal activity of extracts obtained from the leaves of eight selected medicinal plants against different maize seed *Fusarium* pathogens. Extract selection was based on previous in vitro study against the same pathogens [33,34]. Promising extracts were evaluated for their phytotoxicty during seed germination and seedling growth.

## 2. Results

### 2.1. Antifungal Activity against Maize Seeds Inoculated with Fusarium Pathogens

**Figure 1:**
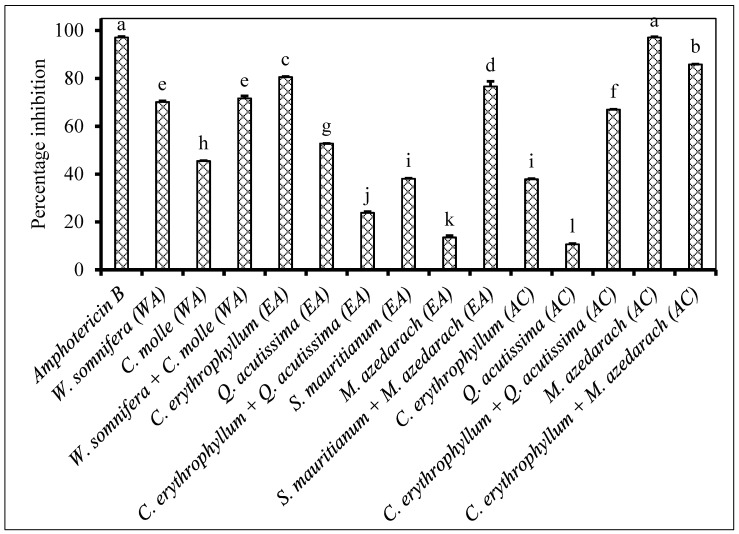
Percentage inhibition by plant extracts against *Fusarium proliferatum* (1 × 10^6^ spores/mL) inoculated on maize seeds. Plant compounds were either extracted in WA: Water, or EA: Ethyl acetate, or AC: Acetone. The extracts were produced from *Withania somnifera*, *Combretum molle*, *Combretum erythrophyllum*, *Quercus acutissima*, *Solanum mauritianum*, *Melia azedarach* and used at a concentration of 2.5 mg dried extract/mL of 10% acetone. Amphotericin B antibiotic (2.5 mg/mL) was used as a positive control. There were 3 replicates per treatment, each comprising 7.5 g of disinfected maize seeds, and the experiment was repeated twice. Data from the two repeat experiments were averaged and analysed statistically. Bars bearing different letters indicate significant differences (*p* = 0.05), as determined by Duncan’s Multiple Range Test.

An individual application of *Combretum erythrophyllum* ethyl acetate, *Quercus acutissima* ethyl acetate and *Melia azedarach* acetone extracts showed antifungal activity of more than 50% inhibition against *F. proliferatum* (Figure 1). However, their corresponding different solvent extractions were less active (≤50% inhibition) against the same pathogen. The combined application of *C. erythrophyllum* and *Q. acutissima* acetone extract showed a somewhat synergistic antifungal activity (66.9% inhibition), against *F. proliferatum*. There was no significant difference in terms of antifungal activity observed between the positive control (97.1% inhibition) and *M. azedarach* acetone extract (97.1% inhibition) against *F. proliferatum* (Figure 1).

**Figure 2:**
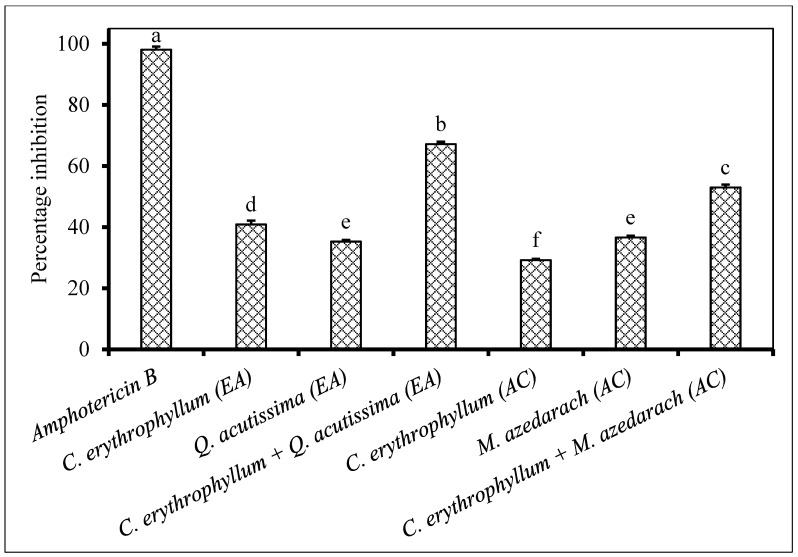
Percentage inhibition by plant extracts against *Fusarium subglutinans* (1 × 10^6^ spores/mL) inoculated on maize seeds. Plant compounds were either extracted in EA: Ethyl acetate, or AC: Acetone. The extracts were produced from *Combretum erythrophyllum*, *Quercus acutissima*, *Melia azedarach* and used at a concentration of 2.5 mg dried extract/mL of 10% acetone. Amphotericin B antibiotic (2.5 mg/mL) was used as a positive control. There were 3 replicates per treatment, each comprising 7.5 g of disinfected maize seeds, and the experiment was repeated twice. Data from the two repeat experiments were averaged and analysed statistically. Bars bearing different letters indicate significant differences (*p* = 0.05), as determined by Duncan’s Multiple Range Test.

**Figure 3:**
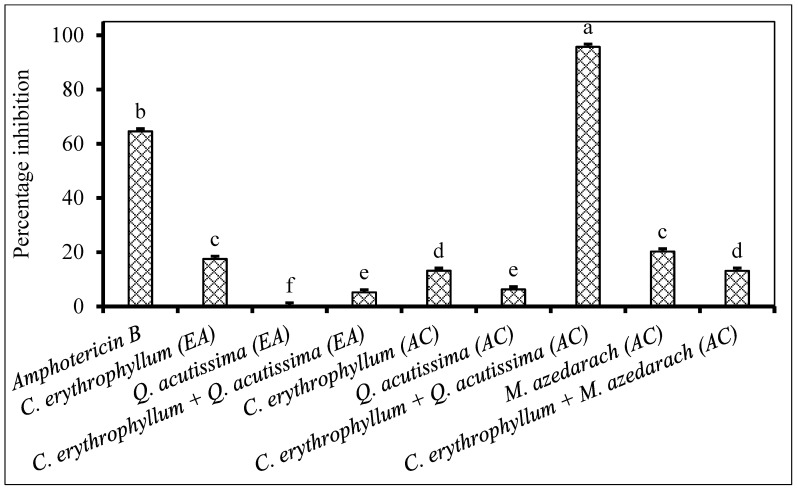
Percentage inhibition by plant extracts against *Fusarium verticilloides* (1 × 10^6^ spores/mL) inoculated on maize seeds. Plant compounds were either extracted in EA: Ethyl acetate, or AC: Acetone. The extracts were produced from *Combretum erythrophyllum*, *Quercus acutissima*, *Melia azedarach* and used at a concentration of 2.5 mg dried extract/mL of 10% acetone. Amphotericin B antibiotic (2.5 mg/mL) was used as a positive control. There were 3 replicates per treatment, each comprising 7.5 g of disinfected maize seeds, and the experiment was repeated twice. Data from the two repeat experiments were averaged and analysed statistically. Bars bearing different letters indicate significant differences (*p* = 0.05), as determined by Duncan’s Multiple Range Test.

Application of individual extracts from *C. erythrophyllum* and *Q. acutissima* demonstrated poor (<50% inhibition) antifungal activity against both *F. subglutinans* and *F. verticilloides* (Figure 2 and Figure 3). The combination of *C. erythrophyllum* and *Q. acutissima* ethyl acetate extract appeared to show a synergistic activity with 67% inhibition against *F. subglutinans* (Figure 2). Similarly, a combination of *C. erythrophyllum* and *Q. acutissima* acetone extracts showed strong, synergistic antifungal activity (96% inhibition) against *F. verticilloides*. Notably, this activity was significantly higher than that of amphotericin B used as a positive control (Figure 3).

**Figure 4:**
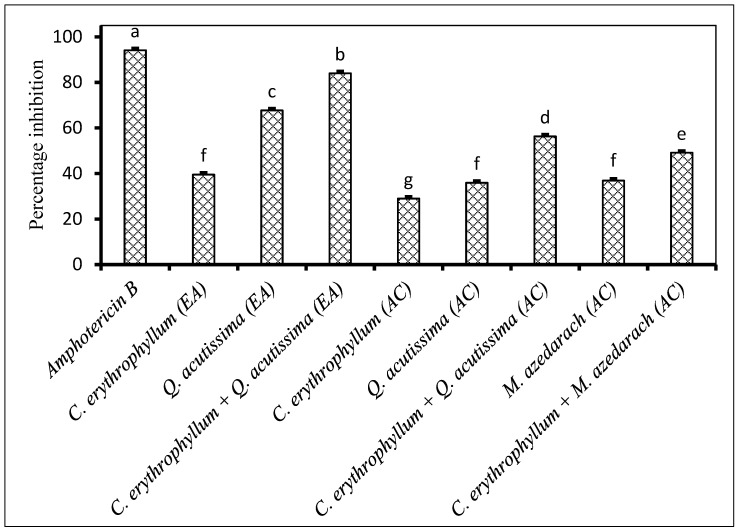
Percentage inhibition by plant extracts against *Fusarium solani* (1 × 10^6^ spores/mL) inoculated on maize seeds. Plant compounds were either extracted in EA: Ethyl acetate, or AC: Acetone. The extracts were produced from *Combretum erythrophyllum*, *Quercus acutissima*, *Melia azedarach* and used at a concentration of 2.5 mg dried extract/mL of 10% acetone. Amphotericin B antibiotic (2.5 mg/mL) was used as a positive control. There were 3 replicates per treatment, each comprising 7.5 g of disinfected maize seeds, and the experiment was repeated twice. Data from the two repeat experiments were averaged and analysed statistically. Bars bearing different letters indicate significant differences (*p* = 0.05), as determined by Duncan’s Multiple Range Test.

Both individual application of acetone and ethyl acetate extracts from *C. erythrophyllum* showed poor antifungal activity against *F. solani* (Figure 4). The combination of acetone or ethyl acetate extracts of *C. erythrophyllum* and *Q. acutissima* resulted in a significantly improved antifungal activity when compared to their individual activity against *F. solani*. Similarly, a combination of *C. erythrophyllum* and *M. azedarach* acetone extracts resulted in a significantly improved antifungal activity (Figure 4).

**Figure 5:**
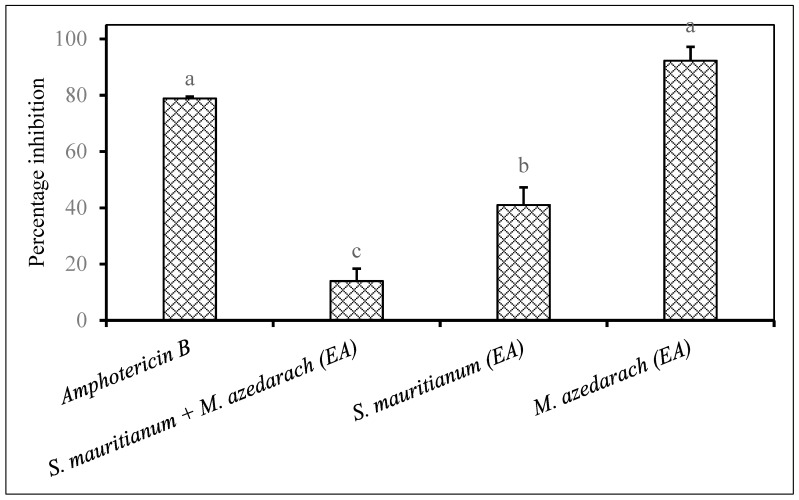
Percentage inhibition by plant extracts against *Fusarium oxysporum* (1 × 10^6^ spores/mL) inoculated on maize seeds. Plant compounds were extracted in EA: Ethyl acetate. The extracts were produced from *Solanum mauritianum*, *Melia azedarach* and used at a concentration of 2.5 mg dried extract/mL of 10% acetone. Amphotericin B antibiotic (2.5 mg/mL) was used as a positive control. There were 3 replicates per treatment, each comprising 7.5 g of disinfected maize seeds, and the experiment was repeated twice. Data from the two repeat experiments were averaged and analysed statistically. Bars bearing different letters indicate significant differences (*p* = 0.05), as determined by Duncan’s Multiple Range Test.

**Figure 6:**
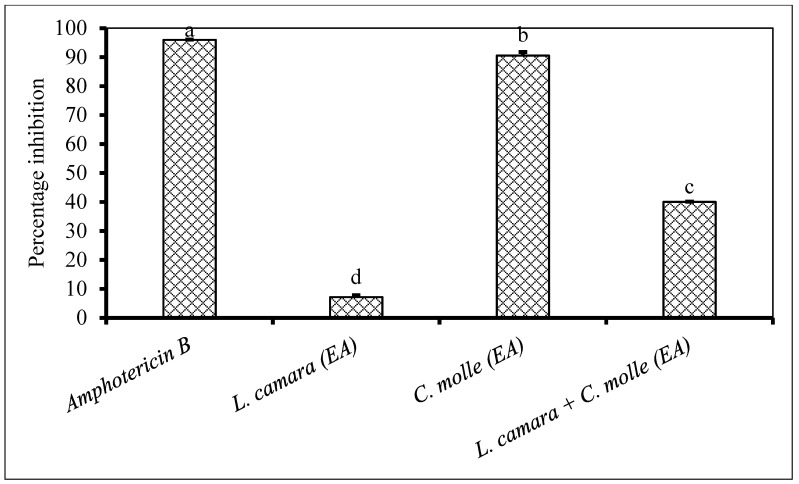
Percentage inhibition by plant extracts against *Fusarium semitectum* (1 × 10^6^ spores/mL) inoculated on maize seeds. Plant compounds were extracted in EA: Ethyl acetate. The extracts were produced from *Lantana camara*, *Combretum molle* and used at a concentration of 2.5 mg dried extract/mL of 10% acetone. Amphotericin B antibiotic (2.5 mg/mL) was used as a positive control. There were 3 replicates per treatment, each comprising 7.5 g of disinfected maize seeds, and the experiment was repeated twice. Data from the two repeat experiments were averaged and analysed statistically. Bars bearing different letters indicate significant differences (*p* = 0.05), as determined by Duncan’s Multiple Range Test.

The combination of *Solanum mauritianum* and *Melia azedarach* ethyl acetate extracts appeared to be antagonistic as the antifungal activity observed against *F. oxysporum* was much less when compared to the individual application of *S. mauritianum* and *M. azedarach* (Figure 5). The same plant extract combination exhibited a synergistic antifungal activity against *F. proliferatum* (Figure 1). The antifungal activity exhibited by *M. azedarach* was equivalent to what was recorded for the positive control (Figure 5). The combination of *Lantana camara* and *Combretum molle* ethyl acetate extract did not improve the antifungal activity against *F. semitectum* when compared to individual application of *C. molle* ethyl acetate, which showed a very strong antifungal activity (Figure 6).

**Figure 7:**
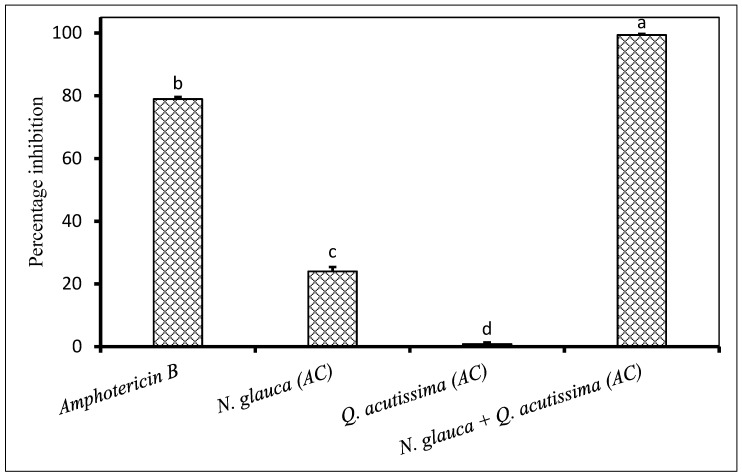
Percentage inhibition by plant extracts against *Fusarium chlamydosporum* (1 × 10^6^ spores/mL) inoculated on maize seeds. Plant compounds were extracted in AC: Acetone. The extracts were produced from *Nicotiana glauca*, *Quercus acutissima*, and used at a concentration of 2.5 mg dried extract/mL of 10% acetone. Amphotericin B antibiotic (2.5 mg/mL) was used as a positive control. There were 3 replicates per treatment, each comprising 7.5 g of disinfected maize seeds, and the experiment was repeated twice. Data from the two repeat experiments were averaged and analysed statistically. Bars bearing different letters indicate significant differences (*p* = 0.05), as determined by Duncan’s Multiple Range Test.

**Figure 8:**
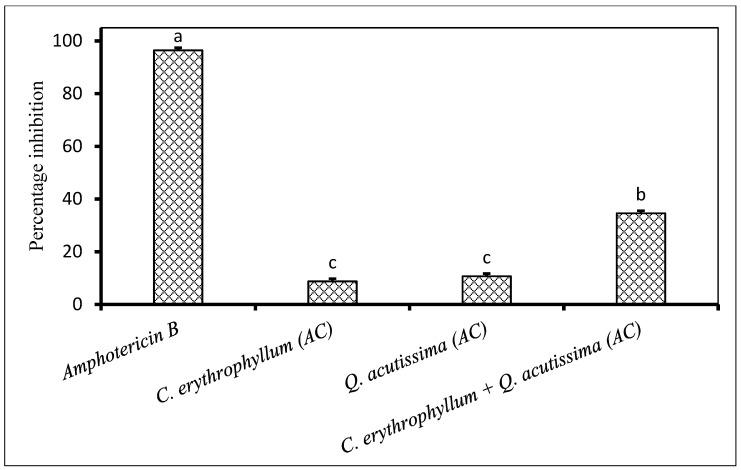
Percentage inhibition by plant extracts against *Fusarium equisite* (1 × 10^6^ spores/mL) inoculated on maize seeds. Plant compounds were extracted in AC: Acetone. The extracts were produced from *Combretum erythrophyllum*, *Quercus acutissima* and used at a concentration of 2.5 mg dried extract/mL of 10% acetone. Amphotericin B antibiotic (2.5 mg/mL) was used as a positive control. There were 3 replicates per treatment, each comprising 7.5 g of disinfected maize seeds, and the experiment was repeated twice. Data from the two repeat experiments were averaged and analysed statistically. Bars bearing different letters indicate significant differences (*p* = 0.05), as determined by Duncan’s Multiple Range Test.

**Figure 9:**
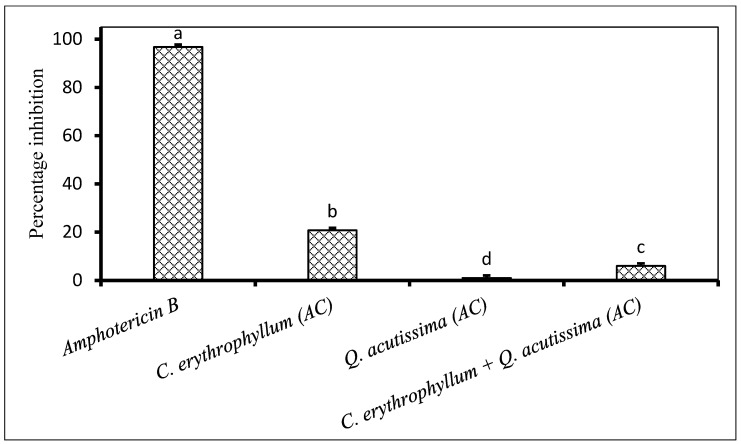
Percentage inhibition by plant extracts against *Fusarium graminearum* (1 × 10^6^ spores/mL) inoculated on maize seeds. Plant compounds were extracted in AC: Acetone. The extracts were produced from *Combretum erythrophyllum*, *Quercus acutissima* and used at a concentration of 2.5 mg dried extract/mL of 10% acetone. Amphotericin B antibiotic (2.5 mg/mL) was used as a positive control. There were 3 replicates per treatment, each comprising 7.5 g of disinfected maize seeds, and the experiment was repeated twice. Data from the two repeat experiment were averaged and analysed statistically. Bars bearing different letters indicate significant differences (*p* = 0.05), as determined by Duncan’s Multiple Range Test.

On the other hand, a combination of *Nicotiana glauca* and *Quercus acutissima* acetone extracts showed synergistic, very strong antifungal activity against *F. chlamydosporum*. This activity was significantly higher compared to that of the positive control (Figure 7). Similarly, the combination of *C. erythrophyllum* and *Q. acutissima* acetone extracts exhibited an improved antifungal activity against *F. equisite* when compared to the antifungal activity of individual extract (Figure 8). The antifungal activity of combined acetone extracts from *C. erythrophyllum* and *Q. acutissima* against *F. graminearum* was 3.5 times weaker compared to the individual application of *C. erythrophyllum* acetone extract against the same pathogen (Figure 9).

### 2.2. Phytotoxicity on Maize Seed Germination

**Figure 10:**
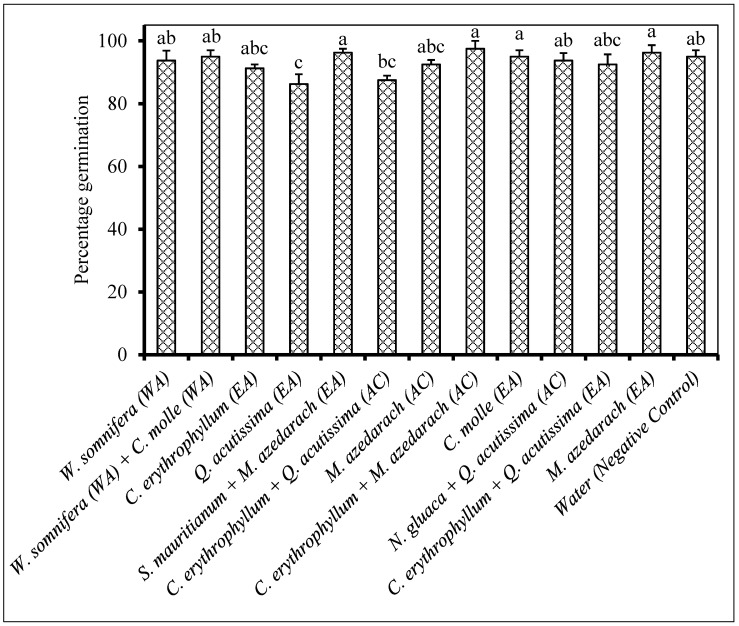
Percentage germination of maize seeds treated with extracts of different plants. Plant compounds were either extracted in WA: Water, or EA: Ethyl acetate, or AC: Acetone. The extracts were produced from *Withania somnifera*, *Combretum molle*, *Combretum erythrophyllum*, *Quercus acutissima*, *Solanum mauritianum*, *Melia azedarach*, *Nicotiana glauca* and used at a concentration of 2.5 mg dried extract/mL of 10% acetone. Water was used as a negative control. There were 5 replicates per treatment, each comprising 20 disinfected maize seeds, and the experiment was repeated twice. Data from the two repeat experiments were averaged and analysed statistically. Bars bearing different letters indicate significant differences (*p* = 0.05), as determined by Duncan’s Multiple Range Test.

Maize seeds treated with *Quercus acutissima* ethyl acetate extract had the lowest percentage germination (86%) as compared to other treatments. There is no significant difference in seed germination of untreated seeds in comparison to all other extract treatments (Figure 10).

### 2.3. Antifungal Activity of Plant Extracts against Pathogen Inoculated on Maize Seedlings

**Figure 11:**
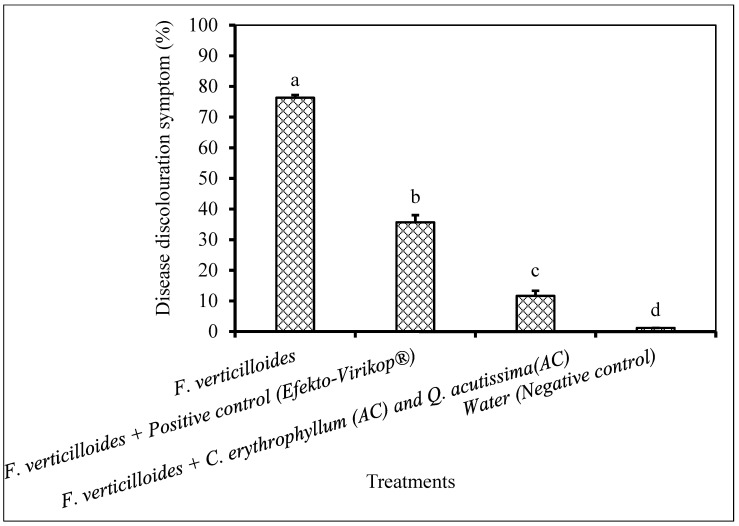
Percentage disease discoloration caused by *F. verticilloides* (1 × 10^6^ spores/mL) inoculated on maize seedlings grown in a greenhouse. Plant compounds were extracted in AC: Acetone. The extracts were produced from *Combretum erythrophyllum*, *Quercus acutissima* and used as combined at a concentration of 2.5 mg dried extract/mL of 10% aqueous acetone. Commercial fungicide (Efekto-Virikop^®^, Efekto, Johannesburg, South Africa) at a concentration of 2.5 mg/mL was used as a positive control and water was used as a negative control. There were 10 replicates per treatment, each comprising one maize seedling per pot, and the experiment was conducted once. Bars bearing different letters indicate significant differences (*p* = 0.05), as determined by Duncan’s Multiple Range Test.

Untreated maize seedlings inoculated with *F. verticilloides* exhibited maximum disease symptom as compared to other treatments (Figure 11). Inoculated seedlings treated with commercial fungicide and plant extract had significantly reduced disease discoloration symptom.

### 2.4. Phytotoxicity on Maize Seedling Growth

Application of *C. erythrophyllum* and *Q. acutissima* acetone extract had no significant effect on the growth of maize seedlings (Table 1). Relative to the control, the extract increased chlorophyll content and dry root mass while stem diameter and number of leaves were reduced.

## 3. Discussion

In this study, we investigated the in vivo antifungal activity of individual and combined plant extracts against *Fusarium* pathogens. Moreover, we conducted the phytotoxicity evaluation of the extracts on maize seed germination and plant growth. We found that, in spite of the polarity of the extraction solvents and the tested pathogens, individual extracts obtained from plant species, such as *W. somnifera*, *C. erythrophyllum*, *Q. acutissima*, *M. azedarach* and *C. molle* showed remarkable in vivo antifungal activity with values ranging from 68% to 98% inhibition.

These plant species are widely distributed in many African countries and are readily available. As an example, *M. azedarach* and *C. erythrophyllum* are often planted as shade or ornamental trees in many human settlements [35]. Therefore, utilization of such plants as bio-pesticides can be achieved at relatively no cost. Management of *Fusarium* species is an important agricultural practice to ensure minimum yield loss during post-harvest storage. Many *Fusarium* species are common causes of maize ear rot diseases and can lead into serious yield reduction in maize farming [16,36,37,38]. They have been frequently isolated in maize production areas both at the field and during post-harvest storage [39,40,41,42]. If not controlled, fungal pathogens including *Fusarium* species can cause about 50%–80% damage on maize grains during storage [39]. In addition, several *Fusarium* species produce mycotoxins that contaminate grains and render them unsuitable for human consumption [18,19,43].

The previous study had shown that combining extracts from different plant species improved antifungal potency in vitro through synergistic and additive interactions, as compared to their individual application [34]. In the current study, a combined application of *C. erythrophyllum* and *Q. acutissima* acetone extract also showed improved antifungal activity against *F. verticilloides*, which was 15 and 7 times stronger when compared to the individual application of *Q. acutissima* and *C. erythrophyllum*, respectively. This combined extract also showed improved activity against *F. solani* and *F. proliferatum*. Nonetheless, a combined ethyl acetate extract from the same plant species (*Q. acutissima* and *C. erythrophyllum*) demonstrated reduced activity against *F. proliferatum* and *F. verticilloides* whereas an improved activity was demonstrated against *F. solani*. The combined acetone extract of *N. glauca* and *Q. acutissima* exhibited an improved antifungal activity against *F. chlamydosporum*. An improvement in the antifungal activity of combined extracts may be due to an increased concentration of antifungal compounds at the inhibition sites on the pathogen. It may also be due to the formation of different chemical constituents with different modes of action. However, not all combined extracts demonstrated an improved activity.

The medicinal plant extracts investigated in the current study did not have a pronounced negative effect on maize seed germination. In smallholder farming, surplus maize seeds and other grains are used for planting and can be recycled for years or generations [5,14]. Therefore, it is essential that plant-based products are not only effective in controlling post-harvest storage pathogens, but they must also not affect seed germination negatively. In a different study, effective botanical insecticide products such as NeemPro^®^ and NeemAzal^®^ (Trifolio-M GmbH Company, Lahnau, Germany) were reported to have shown no negative effect on maize seed germination [44,45]. Furthermore, the current study revealed that a combination of *C. erythrophyllum* and *Q. acutissima* acetone extract showed no toxicity on maize seedling growth. This combination had no significant effect on all seedling growth variables (plant height, number of leaves, chlorophyll content, dry shoot mass, stem diameter and dry root mass) when compared to the negative control. To some extent, this combined extract exhibited in vivo antifungal activity against *F. verticilloides* inoculated on maize seedlings, evident by lower disease percentage area on seedlings inoculated and treated with this combined extract.

Notably, solvent polarity plays an important role in the antifungal activity of medicinal plant extracts. For example, the leaf ethyl acetate extract of *Q. acutissima* was effective (≥50% inhibition) in inhibiting the growth of *F. proliferatum* and *F. solani*. In contrast, acetone extract from *Q. acutissima* was not active against both *F. proliferatum* and *F. solani*. Similarly, the leaf acetone extract of *M. azedarach* was very effective in inhibiting the growth of *F. proliferatum*, while its ethyl acetate extract was ineffective against the same pathogen. The difference in the antifungal activity of acetone and ethyl acetate extracts from the same plant species was also observed with *C. erythrophyllum* against *F. proliferatum*, *F. subglutinans*, *F. verticilloides* and *F. solani*. Acetone is slightly more polar than ethyl acetate [46]. Slight variation in solvent parameter may determine the amount and type of antifungal compounds or metabolites extracted. Medicinal plants contain a wide variety of secondary metabolites including tannins, terpenoids, flavonoids, phenols, saponins and other chemical constituents [47,48,49]. The polarity of the constituent metabolites differs significantly and has influence on their solubility during extraction and thereafter the antifungal activity of the extracts.

Thus, similar to the individual extracts, the activity of the combined extracts also depends on the polarity of extraction solvents and is pathogen-specific. A combination from *C. erythrophyllum* and *Q. acutissima* acetone extract showed improved antifungal activity against *F. verticilloides*, *F. solani* and *F. proliferatum*. Nonetheless, a combined ethyl acetate extract from the same plant species (*Q. acutissima* and *C. erythrophyllum*) demonstrated reduced activity against *F. proliferatum* and *F. verticilloides*, whereas an improved activity was demonstrated against *F. solani*. Apart from polarities of extraction solvents, the difference in cellular structure and defence mechanisms of the pathogens may also influence antifungal efficacy of medicinal plant extracts. It was observed that acetone extract of *M. azedarach* was very effective in inhibiting the growth of *F. proliferatum*, but ineffective against *F. subglutinans*, *F. verticilloides* and *F. solani*. A similar trend was also observed with individual application of ethyl acetate extracts from *S. mauritianum* and *M. azedarach* against both *F. oxysporum* and *F. proliferatum*. *Quercus acutissima* showed large variation in its antifungal activity against different *Fusarium* species (*F. proliferatum*, *F. verticilloides*, *F. chlamydosporum*, *F. solani*, *F. equisite* and *F. graminearum*). Generally, the antifungal activity of the extract appears to depend on both the polarity of extracting solvents and the pathogens.

In vivo antifungal evaluation of individual plant extracts such as *N. glauca*, *L. camara* and *S. mauritianum* did not produce good activity as compared to the results reported in the in vitro study [33]. In other words, these plant extracts showed in vitro antifungal activity with minimum inhibitory concentration (MIC) value of less than 0.1 mg/mL but were ineffective when evaluated in the in vivo study, even at 2.5 mg/mL concentration. This indicates the need to follow up any promising in vitro results with in vivo studies for validation.

The antifungal activity of plants demonstrated in the current study could be due to the presence of different chemical constituents in their leaves. As an example, mollic acid glucoside isolated from acetone leaf extract of *C. molle* was found to be a major terpenoid contributing to the antifungal activity of this plant [50]. Although water and ethyl acetate extracts were used in our study, this terpenoid and other chemicals, such as α-arabinoside and punicalagin, known to be present in *C. molle*, might have contributed to the activity demonstrated by this plant [51]. *Combretum erythrophyllum*, which also showed very good activity, is known to contain antimicrobial compounds such as apigenin, rhamnazin, genkwanin and 5-hydroxy-7,4′- dimethoxyflavane [52]. Hydroxyl-3-methoxcinnamaldehyde, vanillin, scopoletin and pinoresinol isolated from different parts of *M. azedarach* showed good activity against different pathogens including some *Fusarium* species [53,54]. The activity of *W. somnifera* water extract against *F. proliferatum* could be due to withaferin A and withanolide D present in the leaves of this shrub [55,56]. However, efforts are underway to isolate and characterize some actual compounds responsible for the antifungal activity demonstrated by plant extracts in the present study. The extracts with activity greater than 50% inhibition showed potential to control stored maize seed *Fusarium* pathogens. This study demonstrated the ability of botanicals for disease control without any phytotoxicty. Nevertheless, further research is required to establish the phytochemical composition of the extracts, their toxicity against human and livestock, as well as their stability during seed storage, if this method is to be adopted by smallholder farmers.

## 4. Materials and Methods

### 4.1. Collection and Disinfection of Maize Seeds

White maize seeds were obtained from smallholder farmers at Moletjie, ga-Maleka, in Limpopo Province of South Africa. The seeds were collected two weeks after harvest and were visually examined for spoilage, and mechanical and weevil damage. Disinfection of the seeds was achieved by soaking them in bleach (3.5% sodium hypochlorite) for 10 min, followed by rinsing several times with sterilized water and then spraying with 70% ethanol. Thereafter, they were air-dried aseptically in a bio-safety cabinet.

### 4.2. Collection of Plant Materials

Eight medicinal plant species (*Withania somnifera*, *Combretum molle*, *Combretum erythrophyllum*, *Quercus acutissima*, *Solanum mauritianum*, *Melia azedarach*, *Lantana camara* and *Nicotiana glauca*) were selected based on their in vitro antifungal activities against different Fusarium species [33,34]. The leaves of these plants were collected in October and November 2016 from naturally growing plants at the Agricultural Research Council, Roodeplaat, Pretoria and from Capricorn district in Limpopo Province, South Africa. Their identification was confirmed through consultation with Dr. Bronwyn Egan (Larry Leach Herbarium Curator, University of Limpopo) and their voucher specimens were prepared and deposited as previously described by Seepe et al. [33,34].

### 4.3. Preparation of Plant Extracts

Fresh green leaves (about 5.0 kg) were collected into brown paper bags and shade dried immediately at room temperature (25 ± 2 °C). Dried material was grinded into fine powder using a pulveriser (Fritsch Pulverisette 14, Labotec, South Africa) and stored in brown paper bags at room temperature until extraction. Extraction was initiated by adding 100 g of dried powder into an Erlenmeyer flask, followed by addition of 1000 mL solvent. The extraction was done in an ultrasonic bath (Branson, 5510E-MT, Lasec, South Africa) for an hour. The material was extracted separately with each of the three different solvents (water, ethyl acetate and acetone) as previously described by Seepe et al. [34]. The plant material/solvent mixture was filtered through Whatman No.1 filter paper. The residue was re-extracted with equivalent volume of solvent. Ethyl acetate and acetone extracts were separately concentrated using rotary evaporators (Stuart, RE300DB, Lasec, South Africa) and air-dried further in a fume hood. Water extracts were freeze-dried (Sentry 2.0 VirTis SP scientific, United Scientific, Johannesburg, South Africa). All the dried extracts were kept in airtight containers in the dark at room temperature.

### 4.4. Preparation of Fusarium Pathogens

Mycology Laboratory at the Agricultural Research Council - Plant Health and Protection, Roodeplaat in Pretoria provided *F. proliferatum* (PPRI 18,679), *F. semitectum* (PPRI 6739), *F. oxysporum* (PPRI 10,175), *F. subglutinans* (PPRI 6740), *F. chlamydosporum* (PPRI 5116), *F. equiseti* (PPRI 19,029), *F. verticillioides* (PPRI 9278), *F*. *solani* (PPRI 19,147) and *F. graminearum* (PPRI 10,728) used in this study. Fungal strains were sub-cultured on potato dextrose agar (Merck, South Africa) and incubated at 27 °C in the dark for four to seven days. Thereafter, pathogen suspensions were prepared in potato dextrose broth (Merck, South Africa), which were incubated at 27 °C in the dark for three to four days. Fungal spores were collected by straining cultured broth through sterilized cheesecloth. The number of spores was determined using a microscope and haemocytometer, and appropriate dilutions were made to adjust final spore concentration to 1.0 × 10^6^ spores/mL [57,58].

### 4.5. Antifungal Activity of Plant Extracts

#### 4.5.1. In Vivo Activity against Maize Seed Inoculated with Pathogens

Approximately 7.5 g of disinfected maize seeds was added to a sterilized jar, and inoculated with *Fusarium* pathogen (1000 µL) adjusted to 1.0 × 10^6^ spores/mL. The seeds were shaken for few seconds and air-dried aseptically in a bio-safety cabinet for an hour. Thereafter, 1000 µL of medicinal plant extract at 2.5 mg/mL (combined or individual) was added, shaken well and dried for an additional hour. The selection of combined plant extracts was based on their additive and synergistic antifungal activity against tested *Fusarium* pathogens, as was reported in the previous study [34]. In that study, a minimum inhibitory concentration (MIC) value of 0.001 mg/mL was reported; however, some plant extracts have demonstrated antifungal activity of more than 2.5 mg/mL. Hence, the concentration of 2.5 mg/mL was used in in vivo study as the optimal concentration in which the extracts can easily dissolve in acetone without precipitating.

The experiment was repeated twice and each treatment was replicated thrice for every fungal pathogen. Amphotericin B^®^ antibiotic (Phytotek Lab, Pretoria, South Africa) at a concentration of 2.5 mg/mL was used as a positive control. Ten percent aqueous acetone (used to dissolve the dried plant extracts) was included as a negative control. A mixture of seeds and plant extract was included for each treatment and was used for colour or turbidity correction. The jars were sealed and incubated at 27 °C for four days. After incubation period, 20 mL of sterile water was added to each jar and shaken for one minute. The treatments were filtered through cheesecloths and their absorbance measured at 700 nm using Specord 210 spectrophotometer (Analytik Jena, Jena, Germany). Antifungal activity of the extracts was recorded as percentage inhibition of fungal growth, calculated from the absorbance readings of the control and treatment using the following equation:

$$\mathrm{Antifungal}\;\mathrm{activity}\;\left(\%\right)=\left(\frac{C-\left(T-B\right)}{C}\right)\times 100$$

where C is the absorbance reading of the negative control (seeds treated with 10% aqueous acetone and inoculated with fungal pathogen); T is the absorbance reading of the treatment (seeds treated with plant extract or amphotericin B, and inoculated with fungal pathogen); and B is the absorbance reading of the blank (seeds treated with plant extract or amphotericin B only).

#### 4.5.2. In Vivo Activity against Pathogen Inoculated on Maize Seedlings

A combination of *Combretum erythrophyllum* and *Quercus acutissima* acetone extract was evaluated against *F. verticilloides* inoculated on maize seedlings. The extract was selected based on its remarkable antifungal activity exhibited against *F. verticilloides*. Moreover, it showed very strong synergistic antifungal activity against more *Fusarium* pathogens as compared to other combinations as was reported in the previous study [34]. Maize seeds were treated with a combination of *C. erythrophyllum* and *Q. acutissima* acetone extract (2.5 mg/mL) and air-dried in a bio-safety cabinet. Each seed was planted in a 25 cm pot filled with moistened sterilized red soil. The experiment or trial was laid out randomly with ten replications and it consists of untreated seed, plant extract treated seed and commercial fungicide (Efekto-Virikop^®^, Efekto, Johannesburg, South Africa) treated seed. After planting, 200 mL of *F. verticilloides* at 1.0 × 10^6^ fungal spore/mL was added to pots with treated seeds and untreated seeds. The second set of untreated seed received 200 mL water and served as negative control.

The experiment was conducted at the Agricultural Research Council, Roodeplaat Campus, Pretoria, South Africa (latitude 17°49′ S, longitude 31°04′ E), during November/December 2019 and was kept under greenhouse conditions (27.5 ± 2.5 °C and 90.0 ± 5% relative humidity). Four days after planting, 200 mL of combined *C. erythrophyllum* and *Q. acutissima* acetone extract was applied to each of the treatment pots and subsequently every seventh day, while water was added to negative control pots. Commercial fungicide (Efekto-Virikop^®^) at 2.5 mg/mL was also applied to positive control pots at the same interval as plant extract. All the pots were irrigated every second day.

Plant growth parameters (number of leaves, chlorophyll content, stem diameter and plant height) were recorded 20 days after planting and thereafter, were recorded every week. The number of leaves were counted and plant height was measured with tape from soil level to the terminal of the developing leaf. Chlorophyll content was measured from three mature leaves per plant using chlorophyll meter (Minolta, Spad-502, Konica Minolta, Tokyo, Japan) and stem diameter was measured with electronic digital vernier caliper (Calibre MILIM, Digital, Linberts).

After 90 days of planting, the plants were harvested and placed in brown paper bags. A pathogenicity test was also conducted during harvesting period. The crop was cut above soil surface and visually examined for discolouration. Discolouration/disease severity was rated and recorded based on the following scale: 0 = no discolouration, 1 = trace to 25% diseased area, 2 = 25% to 50% diseased area, 3 = 50% to 80% diseased area and 4 = 100% diseased area [59,60]. The root system was carefully removed and soil particles washed off. The harvested materials were oven-dried at 45 °C. At the end of the experiment, data recorded from 20 days after planting to harvesting period were averaged and analysed statistically.

### 4.6. Phytotoxicity Evaluation of Plant Extracts

#### 4.6.1. Maize Seed Germination

Medicinal plant extracts that showed antifungal activity (≥50% inhibition) were evaluated for potential phytotoxicity on maize seed germination. The seeds were soaked overnight in a plant extract at a pre-determined concentration (2.5 mg/mL) and air-dried in a bio-safety cabinet for an hour. Water was used as a control treatment. Twenty seeds were placed per petri dish lined with a moistened double layer filter paper. Each treatment was replicated five times. The experiment was set up in an incubator at constant 25 °C and alternating cycle of 12 h light and 12 h darkness. The filter papers were kept wet throughout the experimental period. The number of germinated seeds was recorded 14 days after sowing. The experiment was repeated twice with five replicates per treatment. Percentage seed germination was calculated using the following equation:

$$\mathrm{Percentage}\;\mathrm{seed}\;\mathrm{germination}=\left(\frac{\mathrm{Number}\;\mathrm{of}\;\mathrm{germinated}\;\mathrm{seeds}}{\mathrm{Total}\;\mathrm{number}\;\mathrm{of}\;\mathrm{seeds}}\right)\times 100$$

#### 4.6.2. Maize Seedling Growth

Maize seeds were treated with a combined *C. erythrophyllum* and *Q. acutissima* acetone extract and air-dried in a bio-safety cabinet. Each treated seed was planted in 25 cm pots filled with moistened sterilized red soil. Untreated seeds were included as negative control. The experiment was conducted at the same greenhouse conditions as detailed in Section 4.5.2 above and it was replicated ten times. Four days after planting, 200 mL of a combined *C. erythrophyllum* and *Q. acutissima* acetone extract (2.5 mg/mL) was applied to each of the treatment pots and subsequently every seventh day, while water was added to control pots. All the pots were irrigated every second day. Plant growth parameters were recorded, averaged and treated as described in Section 4.5.2.

### 4.7. Statistical Analysis

The data was analysed using STATISTICA-8 software. The difference between the treatments for each parameter was evaluated using one-way analysis of variance (ANOVA). Data were expressed as mean ± standard error. Where a statistical significance (*p* = 0.05) was established, means separation was done using Duncan’s Multiple Range Test (DMRT). The difference in growth parameter between negative control and plant extract in the seedling phytotoxicity experiment was analysed using Student’s *t*-test (*p* ≤ 0.05).

## 5. Conclusions

The potential application of medicinal plant extracts as alternative bio-pesticides to protect maize seeds against *Fusarium* pathogens was established. Different solvent extracts from the same medicinal plant species demonstrated different activities against tested organisms. Although some plant extracts can be used individually, the combinations of some extracts exhibited stronger activity than their individual extracts against *Fusarium* pathogens. Almost all the tested extracts did not have any negative effect on maize seed germination. A combination of *C. erythrophyllum* and *Q. acutissima* acetone extract had no negative effect on maize seedling growth. The chemical composition of the extracts and any potential toxic effect on human or livestock, as well as the stability of the extracts during grain storage treatment and the frequency of applications, are aspects that require further research.

## Figures and Tables

**Table 1:** Phytotoxicity of combined *Combretum erythrophyllum* and *Quercus acutissima* acetone extract on maize seedling growth in a greenhouse. Water was used as the negative control. Mean values within rows were not significantly different (*p* ≤ 0.05) according to *t*-test. R.I: relative impact.

Growth Parameters	Negative Control	*C. erythrophyllum* and *Q. acutissima* Acetone Extract	R.I (%)
Plant height (mm)	320.3 ± 34.80 ^ns^	319.8 ± 19.82 ^ns^	−0.14
Chlorophyll content (SPAD)	36.5 ± 2.30 ^ns^	38.0 ± 1.49 ^ns^	4.14
Number of leaves	6.6 ± 0.63 ^ns^	6.2 ± 0.32 ^ns^	−5.85
Dry shoot mass (g)	9.3 ± 1.12 ^ns^	9.2 ± 1.03 ^ns^	−0.19
Stem diameter (mm)	9.85 ± 1.14 ^ns^	8.82 ± 0.58 ^ns^	−10.41
Dry root mass (g)	4.32 ± 1.54 ^ns^	4.55 ± 0.47 ^ns^	5.48

ns: not significant.
